# The pathways between natural disasters and violence against children: a systematic review

**DOI:** 10.1186/s12889-021-11252-3

**Published:** 2021-07-12

**Authors:** Ilan Cerna-Turoff, Hanna-Tina Fischer, Hani Mansourian, Susannah Mayhew

**Affiliations:** 1 Global Health and Development, London School of Hygiene and Tropical Medicine, London, UK; 2 Mailman School of Public Health, Columbia University, New York, USA

**Keywords:** Children, Violence, Child protection, Natural disaster, Humanitarian crisis, Emergency

## Abstract

**Background:**

Natural disasters are increasingly affecting a larger segment of the world’s population. These highly disruptive events have the potential to produce negative changes in social dynamics and the environment which increase violence against children. We do not currently have a comprehensive understanding of how natural disasters lead to violence against children despite the growing threat to human populations and the importance of violence as a public health issue. The mapping of pathways to violence is critical in designing targeted and evidence-based prevention services for children. We systematically reviewed peer-reviewed articles and grey literature to document the pathways between natural disasters and violence against children and to suggest how this information could be used in the design of future programming.

**Methods:**

We searched 15 bibliographic databases and six grey literature repositories from the earliest date of publication to May 16, 2018. In addition, we solicited grey literature from humanitarian agencies globally that implement child-focused programming after natural disasters. Peer-reviewed articles and grey literature that presented original quantitative or qualitative evidence on how natural disasters led to violence against children were included. The authors synthesized the evidence narratively and used thematic analysis with a constant comparative method to articulate pathways to violence.

**Results:**

We identified 6276 unduplicated publications. Nine peer-reviewed articles and 17 grey literature publications met the inclusion criteria. The literature outlined five pathways between natural disasters and violence, including: (i) environmentally induced changes in supervision, accompaniment, and child separation; (ii) transgression of social norms in post-disaster behavior; (iii) economic stress; (iv) negative coping with stress; and (v) insecure shelter and living conditions.

**Conclusions:**

Service providers would benefit from systematic documentation to a high-quality standard of all possible pathways to violence in tailoring programming after natural disasters. The identified pathways in this review provide a foundation for designing targeted prevention services. In addition, the positive coping strategies within certain affected families and communities can be leveraged in implementing strength-based approaches to violence prevention.

**Supplementary Information:**

The online version contains supplementary material available at 10.1186/s12889-021-11252-3.

## Background

Natural disasters are increasingly affecting a larger segment of the world’s population due to climate change and patterns of human settlement [1]. In 2017, the Centre for Research on the Epidemiology of Disasters (CRED) estimated that natural disasters affected 96 million people, and the United Nations Children’s Fund (UNICEF) estimated that natural disasters and other forms of disasters affected approximately 350 million children [2, 3]. Displacement can be considered an indication of extreme exposure to a disaster event. Between 2008 to 2016, an average of 25.3 million people were displaced by natural disasters each year, and although predictions vary, it is estimated that by 2050 extreme weather events will result in forced displacement of over 200 million people [4, 5]. The International Displacement Monitoring Centre (IDMC) estimates that natural disasters caused 18.8 million new displacements in 2017, while armed conflict led to 11.8 million new displacements [6].

Children are considered a priority population in humanitarian response because of their vulnerability to experiencing violence after natural disasters [7]. Natural disasters can disrupt services and societal structures, displace populations, and lead to an increased likelihood of trauma, all of which have been associated with violence in past studies [8–12]. Children may be separated from caregivers or orphaned, leaving them with reduced protection from abuse [13, 14]. In other instances, children may face new vulnerabilities to violence within the home, as their caregivers cope with stressful changes in their environment and threats to their economic stability [15]. Despite a growing number of children affected globally and the implications for public health and development, current understanding is limited as to the full scope of how the social and environmental changes produced by natural disasters may lead to violence against children.

Natural disasters occupy an equivalent status to armed conflict within humanitarian response frameworks and scholarship, and service providers currently implement child protection programming with similar structures, timing, and target populations under a theorical assumption that natural disasters and armed conflict produce identical manifestations of violence against children [16–19]. Structural elements and the affected population’s interpretation of the events may be distinct, however, and as a result, the pathways to violence against children may differ. One of the few studies that modeled family violence among those affected by the Sri Lankan civil war and the 2004 Indian Ocean Tsunami together found that war exposure predicted violence against children (*β* = 0.34, *p* < 0.001), while tsunami exposure acted in the reverse (*β* = − 0.16, *p* < 0.01) [20]. During conflict, the presence of armed actors poses a direct risk for violence which often does not exist in the same manner during natural disasters. Communities and individuals, furthermore, can prepare for certain types of natural disasters, such as typhoons or flooding, that reoccur annually. Indigenous coping mechanisms for managing food supplies and providing social support may reduce the negative impact on human populations [21]. While armed conflict may erode a sense of trust in one’s community and society, a growing body of psychological and sociological research suggests that natural disasters can improve functioning within families and lead to greater sense of community cohesion and altruism [22–27]. Spatial temporal analysis in Chile, for instance, found that social cohesion on the community level increased after large-scale earthquakes and faded over time as conditions normalized [28]. The differential meanings that affected populations ascribe to natural disasters and armed conflicts seem to influence reactions. As a 2014 psychological study on risk judgement illustrates, when people perceive the cause of something as “natural”, they are less likely to judge it as severely as a disaster caused by man [29]. In other words, people respond more negatively to armed conflict than natural disasters, because they perceive natural disasters as outside of human control. This trend is further corroborated in a large-scale review which found that survivors of armed conflict and terrorism had worse mental health outcomes than survivors of natural disasters in samples from 29 countries over two decades [10]. Negative perception and accompanying poor mental health responses may relate to an increased risk of violence against children, as indicated in past studies [30–32]. Greater scholarship on natural disasters and violence against children is needed to begin to decipher potential differences and build child protective services that are specific to natural disasters.

Increasing our understanding of the pathways between natural disasters and violence against children is essential in designing effective violence prevention programs. Service providers have a mandate to provide evidence-based services to prevent any unforeseen harm to children. Identifying the junctures at which one can intervene and the mechanisms by which violence occurs facilitates better tailoring of protection programming. A robust evidence base from stable settings provides helpful insight on factors that can lead to violence against children; however, pathways to violence after natural disasters are less well understood [33, 34]. Elsewhere, we conducted a meta-analysis which showed that there is inconclusive evidence of a direct association between natural disasters and violence against children, but noted that more nuanced research was needed to disentangle pathways to violence [35]. This paper provides a systematic review of peer-reviewed and grey literature to deepen the understanding of the pathways between natural disasters and violence against children and to suggest how this information can be used in the design of future programming.

## Methods

This review adhered to the Preferred Reporting Items for Systematic Reviews and Meta-Analyses (PRISMA) guidelines [36].

### Search strategy

We operationalized the definition of children as people under 18 years of age and physical, emotional, and sexual violence by applying definitions utilized in UNICEF’s *Hidden in Plain Sight* report (refer to Table 1) [37]. Violence prevention falls within the field of child protection, which additionally includes broader issues of neglect and exploitation [19]. These aspects of child protection were not included in this review. Natural disasters were defined as environmental hazards without a direct human cause, as per the conventions of disaster response [38]. We recognize, however, that natural disasters may be spurred by human activities or have distal roots in man-made alterations of the physical environment [39]. We included both slow and sudden-onset natural disasters in this review.

**Table 1:** Operational definitions of violence

Violence form	Definition
Physical violence	“… all corporal punishment and all other forms of torture, cruel, inhuman or degrading treatment or punishment as well as physical bullying and hazing by adults or by other children”
Emotional violence	“Psychological maltreatment, mental abuse, verbal abuse and emotional abuse”
Sexual violence	“… any sexual activities imposed by an adult on a child against which the child is entitled to protection under criminal law” or “… committed against a child by another child if the offender is significantly older than the victim or uses power, threat or other means of pressure”

From: United Nations Children’s Fund [37], p. 4

We searched 15 bibliographic databases and six grey literature repositories from the earliest date of publication to May 16, 2018 (refer to Additional file 1). All searches were restricted to the English language and included all geographic regions. The search strategy applied terms related to three thematic areas: children, natural disasters, and violence (refer to Additional file 2). The search terms were adapted from vocabulary used in previous systematic reviews of children and physical, emotional, and sexual violence and from the national disaster classification categories listed in the Emergency Events Database [40–42]. Grey literature in the humanitarian field tends to take the form of reports based upon rapid needs assessments, regular monitoring of programmatic activities, and evaluations of gaps in service provision. We included any reports, assessments, or evaluations uploaded to the grey literature repositories in initial searches. We solicited additional grey literature from 12 experts within agencies that lead the global child protection response in humanitarian contexts. Focal points whose area of work includes child protection from UNICEF, United Nations High Commissioner for Refugees (UNHCR), International Organization for Migration (IOM), United Nations Population Fund (UNFPA), and International Federation of Red Cross and Red Crescent Societies (IFRC) were contacted. UNICEF focal points, in turn, solicited recommendations for literature from all Child Protection Coordinators and Information Management Officers (IMOs) worldwide. The Child Protection Area of Responsibility (CP AoR)—the global coordination body for child protection in humanitarian contexts led by UNICEF—and IFRC provided supplemental grey literature materials which were not uploaded onto online repositories.

### Selection and analysis

After removal of duplicates, the first and second author independently screened the titles and abstracts of the peer-reviewed articles and the titles and abstracts, executive summaries, and table of contents of the grey literature, as per published guidelines [43]. All peer-reviewed articles and grey literature reports that mentioned both violence against children and natural disasters in these sections were maintained for full text review, after jointly reconciling any conflicting decisions. We used standardized inclusion and exclusion criteria for our decision making in screening (refer to Table 2). A key inclusion criterion was that quantitative, qualitative, or mixed methods sources had to contain original evidence describing information on the pathways between natural disasters and violence against children.

**Table 2:** Inclusion and exclusion criteria

Inclusion criteria	Exclusion criteria
1. Natural disasters are the exposure of interest 2. The outcome measure is any form of violence, including physical, emotional, or sexual violence, bullying, maltreatment, interpersonal violence, or witnessing domestic violence (DV) 3. The person who experiences the violence is a child or adolescent under 18 4. Original quantitative, qualitative, or mixed methods research identifying information on the pathway between natural disasters and violence against children 5. Peer-reviewed articles or grey literature	1. Gang violence, female genital mutilation (FGM), neglect, or child labor, exploitation, trafficking, or marriage as outcomes 2. Editorials, policy reviews or general reports that do not introduce new evidence 3. Conference abstracts 4. Secondary reviews of literature

The full text for 16 grey literature reports was not publicly posted, in which case, we attempted to contact the publication authors directly. No author responded to furnish full texts. We independently double extracted topical information on the disaster and violence context and methodological information on the study design and analysis. We did not place any predetermined criteria on pathway structure. We extracted detailed information on how natural disasters led to violence from qualitative sources. We also extracted the measures between variables in quantitative studies that modeled mediated pathways to violence. If a study used mixed methods, we extracted both qualitative and quantitative information. We solely included information on pathways to violence for those individuals who were below 18 years of age in sources that included acts of violence against adults to abide by our operational definition of children. All information extracted was jointly reconciled by the first and second author. We subsequently conducted a thematic analysis, using a constant comparative method, to sort the data into overarching pathways. The first three authors analyzed themes by jointly discussing how to organize the extracted information from the text into patterns [44]. Using a constant comparative method, the emergent themes were iteratively revised until reaching a consensus that the pathways captured the full meaning of the contained information [45]. The authors’ positionality as insiders in the humanitarian child protection field aided in understanding the lines of demarcation between pathways and supported informed debates on the appropriate organization of information.

### Quality appraisal

We used the Critical Appraisal Skills Programme’s Qualitative Research Checklist and the National Institute of Health Quality Assessment Tools for Cohort and Cross-sectional and Case-Control Study Designs as means of critical comparison (refer to Additional file 3) [46, 47]. In the case of mixed-methods studies, we evaluated the qualitative and quantitative components separately. We positively scored the appropriateness of the article or report’s methodology if it matched at least one of its outlined aims and objectives. The final question in the Critical Appraisal checklist is a subjective determination of value. We rated value based on the article or report's provision of nuanced information and practical recommendations for stakeholders. The research team used these tools in comparing quality, rather than in inclusion and exclusion decisions, which is in-line with the Cochrane Handbook’s guidance for systematic reviews [48].

## Results

### Characteristics of peer-reviewed articles and grey literature

**Fig. 1:**
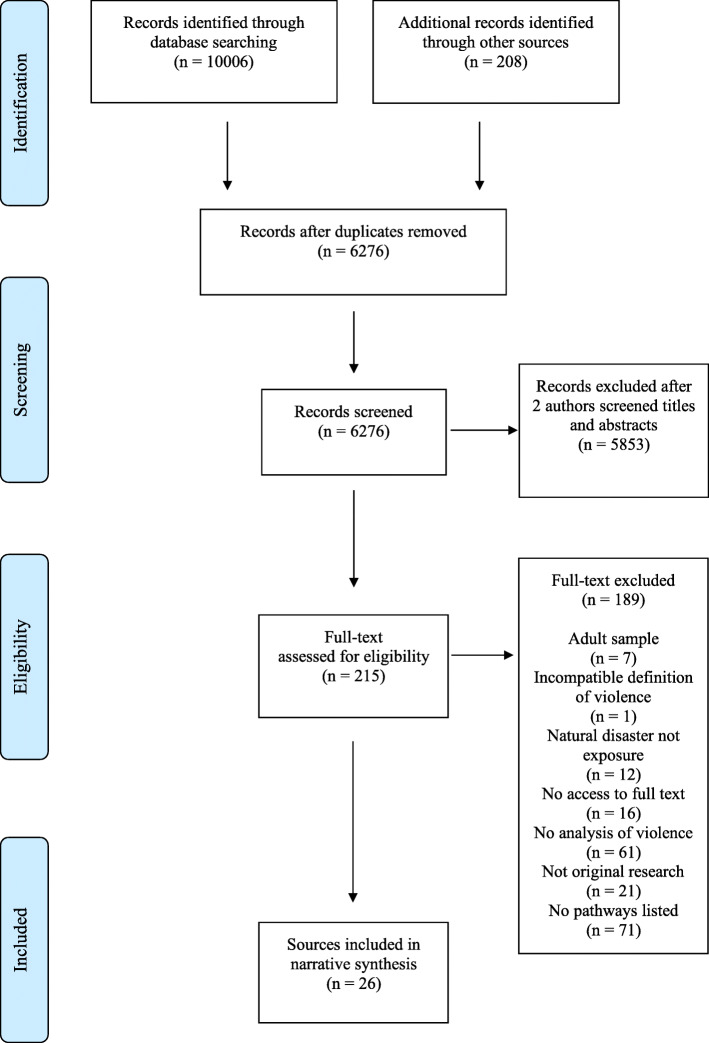
PRISMA flowchart of included sources

We identified a total of 1045 unique peer-reviewed articles and 5231 grey literature publications (refer to Fig. 1). Nine peer-reviewed articles and 17 grey literature publications matched the criteria for inclusion. Amongst the peer-reviewed articles, five of the nine studies utilized qualitative methods, three applied quantitative methods, and one study used both qualitative and quantitative methods. All grey literature used qualitative methodologies.

Two of the peer-reviewed articles described flooding events in 1998 and 2007 in Bangladesh [15, 49], and three publications described four separate disasters in the United States [50–52]. The remaining articles described natural disasters in Haiti, Nepal, and Sri Lanka—two focused on devastating earthquakes [53, 54], and two examined the 2004 Indian Ocean Tsunami [20, 55]. The grey literature described disasters in a range of regions, with the greatest number occurring in Asia and the Pacific and a single report from the Americas on the aftermath of the 2010 Haitian earthquake [56–67]. Drought and extreme tropical storms in the form of typhoons, cyclones, or hurricanes were the most common disasters in the grey literature [56–61, 66, 68–72]. A single report incorporated a temporal element in explaining the pathways to violence; in the Lao People’s Democratic Republic, commonly known as “Laos”, adolescent and adult respondents mentioned that the lack of safety and surveillance and economic hardship in the first one to two weeks after the disaster led to spikes in violence against children that returned to normal levels afterwards [57]. An interview with a Child Protective Services worker as part of a mixed methods study indicated that a possible reverse pattern existed in the United States. He hypothesized that frustration grew over time due to the slow pace of recovery and led to increases in violence against children. The amount of time was not specified, however [50]. Two of the five countries in the peer-reviewed articles and three of the 13 countries in the grey literature were concurrently experiencing armed conflict or some other form of man-made disaster [73].

Sexual violence was the most commonly documented form of violence in the peer-reviewed articles barring Biswas et al. [15], which described physical and emotional violence committed by mothers and fathers against children, and Terranova et al. [51] and Scott et al. [52], which described physical and emotional bullying behaviors among children. In the grey literature, all but three publications contained information on pathways to sexual violence [58, 61, 65]. Physical violence was the second most common form of violence [56, 58, 61, 62, 66–68, 72]. Emotional violence and all forms of bullying were underrepresented in the grey literature [64, 65, 69]. The peer-reviewed literature mainly collected information on children below the age of 18. One article did not specify the exact age range among children under 18 [55], and another recruited respondents as old as 19 years [49]. The grey literature often did not specify the age range of children or used variable age ranges, with some individuals as old as 20 categorized as children [56–58, 61, 66, 68, 69, 71, 72].

### Quality of evidence gathering, analysis, and reporting practices

The peer-reviewed articles and grey literature publications exhibited variable levels of quality in their evidence gathering and reporting practices (refer to Table 3). Six peer-reviewed articles used qualitative methods, namely semi-structured interviews with affected individuals and key informants [15, 49, 50, 53–55]. Four peer-reviewed articles relied upon quantitative surveys that sampled households or school-going children and their parents to collect information on pathways [15, 20, 51, 52]. Overall, the included peer-reviewed articles appropriately analyzed the data. One article, however, attempted to infer quantitative information on incidence from qualitative interviews [55], and another used structural equation modeling which is sensitive to choices in how one analyzes data and relies upon numerous assumptions in its parameters [52, 74]. Within the body of qualitative peer-reviewed publications, several unclearly reported on their data collection methods and did not distinguish between the authors’ views and those of the respondents. Quotes in some instances were extracted without any description of how the information was generated [15], and on the other extreme, information was presented without directly citing respondents in a clear manner [53, 55]. In contrast, Rashid and Michaud’s [49] study of adolescent girls was an example of a nuanced description of violence-related issues after floods in Bangladesh. The study provided explicit analysis of positionality, as Rashid discussed her insider-outsider status as a Bangladeshi who was raised abroad and Michaud’s Canadian identity. Amongst the quantitative peer-reviewed publications, a major threat of bias came from sampling choices. Several studies used samples that were not representative of the entire affected population of children but interpreted them as such. School-going children formed a key group of respondents, but the pathways to violence among those that have access to education may differ from those that do not attend school [20, 51, 52].

**Table 3:** Included peer-reviewed articles and grey literature

					Selected quality markers				
Data source	Country and disaster	Violence type	Age range of children	Co-occurrence of a man-made disaster	Appropriate methodological design	Relationship between researcher and respondents considered	Ethical issues considered	Data analysis rigorous	Risk of bias quality score
Peer-reviewed articles									
Biswas et al. [15]	Bangladesh; 2007 floods	Physical; emotional	Under 18	N	Y	N	N	N (qualitative) Y (quantitative)	4 (qualitative) 8 (quantitative)
Catani et al. [20]	Sri Lanka; 2004 Indian Ocean Tsunami	Physical; emotional; sexual	9–15	Y	Y	N	N	Y	6 (quantitative)
Curtis et al. [50]	United States; 1989 Hurricane Hugo; 1989 Loma Prieta Earthquake; 1992 Hurricane Andrew	Physical; emotional; sexual	Under 18	N	N	N	N	N	1 (qualitative)
Davis and Bookey [53]	Haiti; 2010 earthquake	Sexual	5–18	N	Y	N	N	N	2 (qualitative)
Fisher [55]	Sri Lanka; 2004 Indian Ocean Tsunami	Sexual	Not reported	N	N	Not reported	Not reported	Not reported	3 (qualitative)
Rashid and Michaud [49]	Bangladesh; 1998 floods	Sexual	15–18	Y	Y	Y	Y	Y	8 (qualitative)
Scott et al. [52]	United States; 2005 Hurricane Katrina	Emotional (bullying)	8–15	N	Y	N	Y	Y	9 (quantitative)
Standing et al. [54]	Nepal; 2015 earthquake	Sexual	Under 18	N	Y	N	N	N	3 (qualitative)
Terranova et al. [51]	United States; 2005 Hurricane Katrina	Physical and emotional (bullying)	Fifth graders (mean: age 10)	N	Y	N	N	Y	8 (quantitative)
Grey literature									
CARE Ethiopia Emergency Unit [71]	Ethiopia; 2015-present drought	Sexual	Not reported	N	Y	N	N	N	5 (qualitative)
Child Protection Sub-Cluster [56]	Philippines; 2012 Typhoon Bopha	Physical - younger children; sexual - in households	Not reported	Y	N	Not reported	Not reported	N	5 (qualitative)
Civil Protection Zimbabwe [70]	Zimbabwe; 2017 Tropical Cyclone Dineo	Sexual (particularly ages 5–18)	Under 18	N	Y	N	N	N	5 (qualitative)
Government of Bangladesh and Humanitarian Coordination Task Team of Bangladesh [67]	Bangladesh; 2017 floods	Physical; sexual	Under 18	N	Y	N	N	Y	8 (qualitative)
International Federation of Red Cross and Red Crescent Societies [57]	Laos, Indonesia, and Philippines; 2016 Oudomxay Floods and 2009 Typhoon Ketsana in Sekong; 2016 Aceh Earthquake and 2016 Aceh and Bima Flash Floods; 2013 Typhoon Haiyan	Sexual	Not reported	Y (Philippines)	Y	N	N	Y	7 (qualitative)
Jinks and Komenji [58]	Papua New Guinea; 2016 frost and drought	Physical	Not reported	N	Not reported	Not reported	Not reported	N	1 (qualitative)
Ministry of International Affairs of the Government of the Kingdom of Tonga and Pacific Humanitarian Cluster [61]	Tonga; 2014 Cyclone Ian	Physical	Not reported	N	Y	Not reported	Not reported	Not reported	6 (qualitative)
Oxfam and CARE Ethiopia [69]	Ethiopia; 2015-present drought	Emotional; sexual	Not reported	N	Y	N	N	Y	8 (qualitative)
People in Need Czech Republic [62]	Nepal; 2015 earthquake	Physical; sexual	Under 18	N	Y	N	N	N	6 (qualitative)
People in Need Czech Republic [63]	Nepal; 2015 earthquake	Sexual	School-aged girls, grades 6–10	N	Y	N	N	Y	6 (qualitative)
Plan International, Save the Children, United Nations Children’s Fund and World Vision [64]	Nepal; 2015 earthquake	Emotional; sexual	8–12 and 13–18	N	Y	N	N	Y	8 (qualitative)
Polack [59]	Kenya; 2006–2009 drought	Sexual	Under 18	Y	Y	N	N	N	4 (qualitative)
Save the Children [65]	Mongolia; 2016–2017 dzud	Bullying	6–16 in FGD and not reported	N	Y	N	N	N	4 (qualitative)
Save the Children [72]	Somalia; 2015-present drought	Physical; sexual	Not reported	Y	Y	N	N	N	6 (qualitative)
Save the Children [66]	Papua New Guinea; 2014–2015 drought	Physical; sexual	Not reported	N	Y	N	N	N	6 (qualitative)
Save the Children [68]	Kenya; 2011–2012 East Africa drought	Physical; sexual	10–11 and 12–16 in FGD and not reported	Y	Y	N	N	N	4 (qualitative)
United Nations Population Fund and Ministère à la Condition féminine et aux Droits des femmes [60]	Haiti; 2016 Hurricane Matthew	Sexual	15–18	N	Y	Not reported	N	N	4 (qualitative)

*Y* Yes and *N* No. In the case that a dataset included people over the age of 18, the age range indicates the viable sample of respondents under the age of 18 that was utilized for analysis. The Uppsala Conflict Data Program [73] was referenced to confirm the presence of man-made disasters in the same country, region, and timeframe

The grey literature applied a combination of qualitative methodologies, including interviews with directly affected communities, focus group discussions (FGD), direct observation, and key informant interviews. All studies appropriately sought out both female and male respondents to capture gendered perspectives of pathways and separated FGD by gender. Notably, most of the grey literature directly engaged children as respondents, and two reports partitioned children into separate age ranges [64, 68]. The chosen methodologies, however, were not described in detail, and reporting was minimal at best. The grey literature treated the evidence as if it was representative of the entire affected population and did not explore limitations or differences in perspectives. Some reports did not include an abstract or overview, while others did not provide complete information on the research questions, methodologies, and findings. Within the main body of text, only one report provided details on how the authors synthesized evidence [64]. The presentation of the findings on pathways was similarly presented without sufficient depth. It was often impossible to ascertain the extent to which the description of the pathway constituted the opinion of the respondents or a secondary interpretation by the publication authors. The extent to which local researchers led the production of knowledge, likewise, was challenging to assess. Four publications indicated that they were written or led by international researchers from outside of the disaster country [57, 59, 62, 63]; two others implied that international teams had a significant role in the production of knowledge [56, 60]; and seven additional publications were spearheaded by the national arm of an international non-governmental organization [64–66, 68, 69, 71, 72]. A governmental agency was the first author for three reports from Tonga, Zimbabwe, and Bangladesh [61, 67, 70].

### Pathways between natural disasters and violence against children

The pathways identified in the literature were thematically organized into five categories: (i) environmentally induced changes in supervision, accompaniment, and child separation; (ii) transgression of social norms in post-disaster behavior; (iii) economic stress; (iv) negative coping with stress; and (v) insecure shelter and living conditions.

### Environmentally induced changes in supervision, accompaniment, and child separation

Respondents identified changes following natural disasters in caregiver and children’s travel and movement which produced new patterns of accompaniment and separation. In some settings, the changes increased violence, and in others, they were protective. In Ethiopia, for instance, two grey literature reports identified new gendered patterns of movement that increased sexual violence risk. During the drought, girls ventured further away from their homes to find water or were left in their households alone for long time periods while their mothers fetched water. As water sources became scarcer, girls began to collect water at night to avoid queues during the day. Male family members, likewise, were forced to migrate longer distances to find paid work and tend to their cattle, which left female members of the household alone [69, 71]. Each of these changes—movement to isolated locations, being left unaccompanied in the home, and travel in darkness—increased the risk of violence against girls. Findings from droughts in Kenya and Somalia similarly reinforced that girls were at risk of sexual violence when they searched for food, firewood, and water or travelled for work, particularly in the early morning or evening [59, 72]. Street harassment was common, and although respondents admitted that it had existed prior, “It was more scary during the floods because there were more *mastaans* [hoodlums] and *goondahs* [thugs] hanging about” (Rashid and Michaud [49], p. 62). This increase in unknown men congregating in a new shared space created a situation ripe for opportunistic sexual violence. Women and girls increasingly relied on physical proximity to their former neighbors and communities or on sending girls to stay with distant family members as a means of protection. In contrast, boys experienced distinct forms of violence as they travelled. Respondents believed that boys who moved to towns and away from their homes in Somalia during a drought faced heightened risk of physical violence from employers and other adults [72]. Violence between children additionally increased in some settings. In Mongolia, adults from herder communities spent a greater amount of time tending to cattle during extreme winter conditions, which led to minimal supervision and increases in bullying from peers [65].

Permanent separation from friends and adult caregivers produced some of the greatest ongoing sexual violence risks, especially for girls. Girls who lived alone in camps after the 2010 Haitian earthquake and after the 2015 Nepal earthquake faced sexual violence from strangers, as spaces were often transitory and lacked strong systems of policing and social control [53, 54]. Respondents in one study believed that girls whose mothers died during the 2004 Indian Ocean Tsunami in Sri Lanka were at risk of sexual violence within their homes from fathers, brothers, or other male family members. Risk was attributed to the isolation of girls with male family members and the reduced ability of adult members of the household to have sexual intercourse, given the lack of privacy [55]. The provided reasons are proximal, however, and do not analyze problematic aspects of gender norms which dictate that spaces must be gender segregated to prevent sexual violence and that men and boys cannot control sexual desires, notwithstanding the societal taboo of incest.

In contrast, a series of grey literature reports described how changes in travel and movement protected children from violence. After the 2015 Nepal earthquake, respondents mentioned that parents became more protective of their children and restricted their mobility, leading to less violence from strangers [64]. The reason why a shift occurred in parenting and how it intersected with parents’ past disaster experiences was not explored. In a report on a Kenyan drought, some respondents believed that sexual violence against girls decreased, because girls who would have faced risks while herding were now either in school or working as domestic helpers since the cattle had largely died [68]. Two grey literature reports from the Pacific region highlighted culturally-specific coping mechanisms that prevented violence after natural disasters [61, 66]. Although not dissected further, communities in Tonga outlined that traditional values led to the creation of community safety nets after a cyclone so that adults in the communities watched children outside of their homes and prevented any acts of violence [61]. Respondents in Papua New Guinea similarly mentioned that joint family structures were protective against sexual violence during a drought, because young children were not left alone, and children travelled long distances in groups to fetch water [66].

### Transgression of social norms in post-disaster behavior

Natural disasters lead to structural changes, and individuals may adapt their behaviors in ways that transgress social norms. A clash of meaning exists when individuals interpret behavior by what is deemed “normal” in stable settings. This signification of behaviors as transgressive may cause individuals to act out in violent ways against children. In Nepal, it is traditionally believed that women and girls are “unclean” when they menstruate, but girls had limited access to basic feminine hygiene products after displacement from flooding. They commonly slept outside of their tents to avoid “polluting” the household and as a result, experienced sexual violence from strangers [54]. Similarly, in Bangladesh after floods, adolescent girls mentioned that they faced sexual harassment *en route* to work, because by wading through flood waters, it caused the fabric of their saris to cling to their bodies in a socially unacceptable manner [49]. In the Afar region of Ethiopia, some communities practice a tradition known as *mira*, or entitlement for men to have forced sex with women and girls while their husbands are away. *Mira* increased the rape of married women and girls as male members of the household migrated further and for longer periods of time to find work during a drought [71]. Another example from Somalia involves increases in physical violence against boys. Harmful gender norms dictate that boys are expected to generate income for the family. If boys failed to support their families, caregivers considered physical violence as merited despite the constriction of economic opportunities during a drought [72]. These acts of violence occurred after natural disasters, but normative beliefs and attitudes about physical violence often preceded the disaster event, as underscored in focus groups of men and women in Bangladesh following the 2017 floods [67].

### Economic stress

The economic stress of natural disasters especially affects households living in poverty [15, 57, 62]. One proposed reason is that men, frustrated by economic loss and hardship, misdirect their anger at sexual partners and children. Men identified insufficient cash assistance after the 2015 Nepal earthquake and economic loss from not harvesting crops before floods in Laos as reasons for why they were physically violent [57, 62]. After flooding in Bangladesh, men who received aid or took out personal loans were more than twice as likely to be physically or emotionally violent with their children than those who could rely on personal savings (adjusted odds ratio [aOR]: 2.06, 95% CI: 1.08–3.95, *p* < 0.01 and aOR: 2.62, 95% CI: 1.45–4.74, *p* < 0.001, respectively) [15]. Financial instability, loss of income generating activities, and economic reliance on others seemed to elicit a similar violent reaction among men. The deeper structural drivers relate to gender norms that dictate that men are supposed to be economic providers and are entitled to act out their emotions on women and children. However, the trigger for the behavior was inextricably linked to economic stress and loss caused by natural disasters.

Women were likewise affected by economic stress. In Sri Lanka after the 2004 Indian Ocean Tsunami, decreases in economic status predicted violence from both mothers and fathers (*β* = − 0.20, *p* < 0.001) [20]. Mothers in Bangladesh were the member of the household that was most often physically violent against children, and those who did not generate an income outside of their households were 3.53 times (95% CI: 1.67–7.46) more likely to abuse their children emotionally or physically [15]. An interview with a father from the study describes why caregivers were physically abusive after economic loss, “My child asked me in the morning to bring back cookies when I went outside to search for work. It was happening sometimes when we could only afford to eat once a day, so how could I buy cookies? I couldn’t control myself and I slapped the child” (Biswas et al. [15], p. 6). Children could not understand the financial strain on their households, and in demanding superfluous goods, it triggered caregivers to lash out in frustration and guilt. In addition, children were occasionally physically violent against each other due to economic stress. After Typhoon Bopha in the Philippines, a minority of older children were physically abusive against their younger siblings as a result of competition for limited food and the stress of confined living quarters [56]. Girls, in particular, face physical and sexual violence risk outside of their homes. Economic hardship pushes women and girls into precarious employment where work relationships are often exploitative. Two reports specifically mentioned that employers took advantage of the increased vulnerability and power imbalances to abuse their underage female employees physically and sexually [57, 68].

### Negative coping with stress

The literature documents two ways by which negative coping after natural disasters leads to violence against children. First, a proportion of men respond to natural disasters by abusing substances and gambling, which exacerbates sexual and physical violence [54, 62, 63]. Evidence from a study in Sri Lanka post-tsunami indicated that fathers’ alcohol use was a significant factor associated with committing physical, emotional, or sexual abuse against their children (*β* = 0.16, *p* < 0.01) [20]. Similarly, after the 2015 earthquake in Nepal, men negatively coped with stress by purchasing and consuming greater amounts of alcohol despite a three-fold increase in price. Alcohol abuse emboldened men to commit acts of sexual violence against girls in the community [62]. Violence against women by their partners may have further ripple effects on children. For instance, women in Bangladesh after floods who experienced physical, emotional, or sexual violence from their husbands were nearly five times more likely to abuse their children than those who were not (aOR: 4.53, 95% CI: 1.94–10.60) [15].

Second, caregivers reported that they had less patience for children during a drought in Ethiopia and after flooding in Bangladesh and as a result, would more frequently and disproportionately chastise their children when asked questions [15, 71]. The phenomenon is poignantly captured in the words of a respondent, “I can’t stop my emotions during a devasting situation. My 6 years old child always wants to know about this and about that and it disturbs me. So I say something bad to my child …” (Biswas et al. [15], p. 6). Caregivers’ capacity to regulate their anger was eroded by the stress of the disaster, and they misplaced their anger on their children. The inability of adults to regulate their anger also affects children outside of their immediate households. In Papua New Guinea, hungry children who stole vegetables from neighboring plots were repeatedly beaten [58].

An extreme stress reaction to natural disasters can manifest as post-traumatic stress disorder (PTSD) for a minority of the population [75, 76]. The literature identified in this review, however, yielded inconclusive evidence on the pathway between natural disaster exposure, PTSD, and violence. Using structural equation modeling, Scott et al. [52] found that the relationship between exposure to Hurricane Katrina in the United States and emotional bullying was completely mediated by PTSD. The finding contrasted with hierarchical modeling which revealed that PTSD did not predict emotional or physical bullying after Hurricane Katrina [51].

### Insecure shelter and living conditions

The safety of shelter and living conditions directly relates to a risk of sexual violence against children after natural disasters [77]. Respondents mentioned several elements of inadequate shelter construction and logistical management that increased risk, including: the ease of entry when flimsy tarp materials were used for temporary housing or the inability to lock housing structures; a lack of privacy due to the design or incomplete construction of shelters which allowed men to see girls while changing clothing; and most commonly, the overcrowding of unknown families into the same living space [53, 54, 57, 60, 63, 64, 70]. Rashid and Michaud’s [49] study of the effects of flooding on adolescent girls in Bangladesh described shelter risks that were particular to floods. Adolescent girls were forced to sleep on rooftops due to the submersion of the lower levels of their homes. Sleeping outside produced vulnerability to sexual violence from strangers at night. Girls, furthermore, were increasingly exposed to unknown men while conducting their daily activities outside of the household, such as bathing and using latrines. As was the case with housing, the structure of the bathing and toilet facilities may increase the risk of sexual violence. The spaces and structures had a general inadequacy of lighting and were not gender segregated or securely locked [49, 53, 57, 62]. One report nuances the discussion by stating that although structural insecurity existed in communities prior to natural disasters, the events exacerbated safety risk by limiting the mobility of women and girls [67].

## Discussion

We identified multiple pathways between natural disasters and violence against children. Each pathway presents a meaningful juncture to intervene in preventing violence. It is promising that many interventions already exist that can be implemented or adapted, and the expertise and operational structure do not need to be built anew; for example: SASA! for norms change [78], Parents Make the Difference for positive parenting [79], and Cure Violence for creating safe environments [80], to name a few. In addition, global guidance, as outlined in the *Minimum Standards for Child Protection in Humanitarian Action (CPMS)* and the World Health Organization’s (WHO) *INSPIRE: Seven Strategies for Ending Violence against Children*, provide standards that should be met in building key components of interventions [19, 81]. Service providers would benefit from linking programmatic activities to pathway structures and ensuring robust coordination across agencies to address all possible paths to violence. As an example, cash transfers for families via male caregivers may alleviate economic stress but may also increase violence against children without changing gender norms that stigmatize men for not being able to provide for their families economically. Another concurrent pathway may lead to violence by way of negative coping with stress. Interventions to prevent violence against children in this instance would therefore need to be multi-pronged and change community norms, provide psychosocial support, and reduce problematic substance use to be effective. Alternative provision of cash transfers to female caregivers would still likely lead to violence against children without intervening on normative gender roles with their male partners and providing psychosocial support and parenting interventions for women. Overall, identification of the underlying pathways to violence against children aids in making decisions about programmatic structure more intentional and targeted.

Economic stress and negative coping with stress were identified as two important pathways to violence against children in this review. It is unclear if investment should be equal across all pathways, however, and further research should compare the relative importance of these pathways across natural disaster contexts. It is likely that many pathways are still unknown and should be identified to improve the effectiveness of programmatic design. Pathways to violence may likewise differ by violence type. The majority of studies captured information on sexual violence which is unexpected, given that physical and emotional violence against children are often more prominent measures in the field of child protection [82]. Comprehensive mapping is needed to decipher how pathways may differ for each specific form of violence. Furthermore, this review indicated that pathways between natural disasters and violence against children are indirect. An analysis of the effect of natural disasters on violence, therefore, may mask the underlying relationship without taking mediating factors into account [35, 83]. Future evidence production would benefit from measuring co-occurring factors and accounting for the timing of each element on the pathway between a natural disaster event and violence against children.

Nuanced information on pathways is key in understanding how natural disasters lead to violence against children. The evidence base needs greater documentation of how violence differs across settings, by natural disasters type, and in instances where concurrent man-made disasters exist [35]. These differences likely have major implications for violence outcomes. Larger questions remain about whether armed conflict and natural disasters share all pathways to violence. This review did not identify radically different mechanisms. The single study from Sri Lanka that directly compared exposure types, however, found that natural disasters reduced levels of physical and emotional violence in households, whereas armed conflict increased these forms of violence [20]. Several grey literature sources in this review further highlighted that families and communities exhibited protective behaviors after natural disasters which respondents attributed to reductions in violence [61, 64, 66, 68]. Although tentative, the evidence suggests that violence against children may not always increase after natural disasters; that armed conflict and natural disasters may act differently to produce different violence patterns; and that certain positive coping behaviors may successfully moderate or prevent violence after natural disasters [50]. Greater research is needed, therefore, to identify why differences may exist and which factors support the development of protective behaviors. Moreover, given the overarching evidence in this review that multiple pathways to violence exist after a natural disaster event, a better understanding of attributes and behaviors that prevent violence is paramount. In particular, the current body of academic research could benefit from a more comprehensive approach in documenting which indigenous strategies have been successfully implemented after natural disasters. Academic research should capture information on individual strengths and protective behaviors, rather than solely factors that increase vulnerabilities to violence. Furthermore, pathways to violence may differ between developed and developing countries. A 20-year study of mental health after natural disasters found that people in developing countries faired far worse than those in developed countries. The authors’ suggested that individuals have negative mental health outcomes when they knew that they could not access social services [10]. The implication is that individuals living in developing countries are potentially at greater risk of committing violence against children after natural disasters, given worse mental health indicators on the individual level, but also, are at higher risk because social safety nets and systems of protection are often not robust [84].

The impact of natural disasters is likely uneven across populations. Gender is an important axis of difference which was not thoroughly explored in the peer-reviewed articles and grey literature. Girls and boys experience sexual violence at different levels in stable settings, and this dynamic may be reflected in natural disasters [85]. Past research has found that people with lower education and minority populations receive less social support in disaster recovery, which impacts the ability to cope with an overwhelming situation [86]. Although not a direct measurement, a recent longitudinal study from the United States confirmed that particularly African Americans and Hispanics, individuals with lower levels of education, and those who did not own homes were less likely to recover economically from natural disasters, and in fact, natural disasters entrenched wealth inequalities further [87]. Considering the clustering and intersectionality of poverty, limited educational opportunities, and race and ethnicity, it is probable that natural disasters compound already existent vulnerabilities in specific groups [88, 89].

Our understanding of pathways between natural disasters and violence against children hinges upon the quality of humanitarian evidence gathering and reporting. Much of the information in this review, particularly within the qualitative studies and grey literature, did not present information in a standardized or comprehensive manner, which hinders cross comparison and meaningful interpretation. Greater documentation of methods is needed to enable the reader to understand how the data was collected and assess the accuracy of the author’s description of pathways. In both qualitative and quantitative studies, the study population should correspond with the research question. The literature base would benefit from interrogating which segment of the overall population of children is represented in each study and its appropriateness; how these choices lead to identification of different pathways to violence; and which biases exist in reporting information, given the positionality and identity of data collectors in relation to the affected population and the authors’ approach in synthesizing information.

**Fig. 2:**
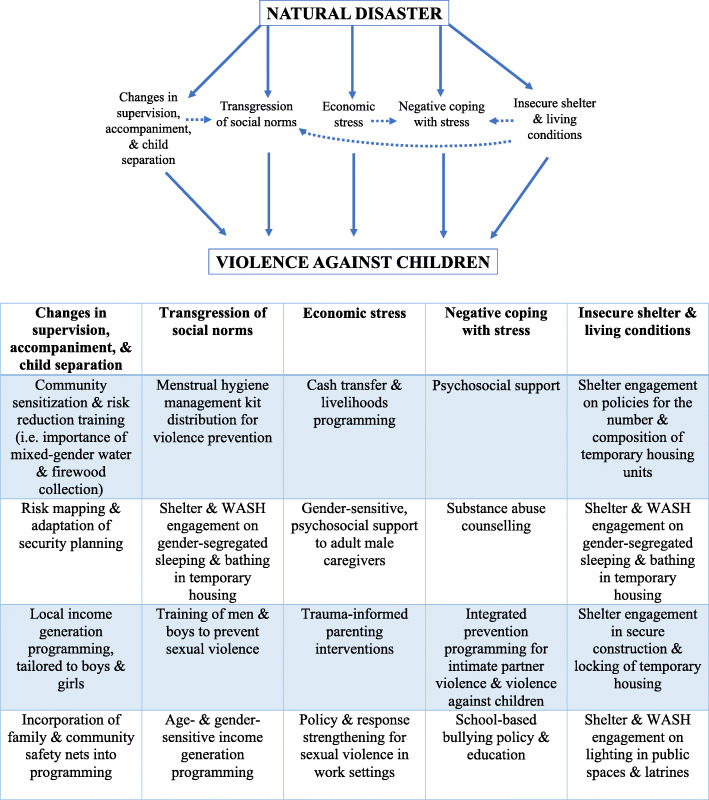
Pathways to violence against children and potential interventions by pathway type. WASH refers to the Water, Sanitation, and Hygiene sector and programming of a humanitarian response

Despite the limitations of the existing literature, it is possible to draw a tentative mapping of the likely pathways to violence and possible points of intervention that service providers should consider when designing their programming. The mapping outlined presents a starting point in identifying viable points for intervention and creating programmatic structures to prevent violence against children (refer to Fig. 2).

### Strengths and limitations

We presented a detailed examination of peer-reviewed articles and grey literature on the pathways between natural disasters and violence against children. We extensively reviewed grey literature, which is often neglected in systematic reviews but is the main body of evidence in the humanitarian field. We included all forms of natural disasters globally, particularly in low- and middle-income countries. Our review, thus, contributes to a better understanding of the research gaps and programmatic opportunities for protecting children from violence in the wake of natural disasters. The review was limited by inconsistent posting of grey literature in online repositories. We examined the prominent grey literature sites for child protection in humanitarian settings, supplementing our search with targeted outreach to key international bodies that lead response efforts. However, we likely excluded sources that were not posted on online clearing houses or were not available in electronic form. Grey literature reports frequently are not shared publicly and so, would not have been identified. As an English language review, we may have missed a subgroup of articles and reports written in non-English languages.

### Implications for improving future practice

Child-focused programming after natural disasters is often designed to address isolated pathways to violence against children. Our findings illustrate the need to design programming that responds to multiple pathways. Protection interventions that address a single pathway to violence are likely to prove ineffective, because other routes to violence against children continue to exist. A comprehensive mapping of all potential pathways to violence against children after natural disasters would allow for individual agencies to better tailor their programmatic design to key upstream drivers of violence and for coordination bodies to identify any gaps in response efforts. Isolated peer-reviewed articles and grey literature publications importantly identified cultural and location-specific coping strategies that were protective. Families and communities may possess indigenous knowledge that reduces the negative impact of natural disasters and aids in protecting children from violence [61, 66]. As a result, natural disasters may offer opportunities to structure child protection interventions to support and bolster local response efforts. The approach has implications in terms of effectiveness (i.e., building upon existent prevention strategies is easier than promoting strategies seen as externally enforced or outside of societal norms), financing, and paradigm shifts to localize response after natural disasters in line with global commitments, such as the Grand Bargain for humanitarian financing [90]. This review, likewise, highlights our need for greater documentation, given the paucity of sources, and higher quality information for future investigation and intervention.

## Conclusions

As natural disasters increasingly affect human populations, service providers need to better understand the pathways between natural disasters and violence against children. The pathways identified in this systematic review highlight specific elements of the post-disaster environment that can be leveraged or targeted to create effective interventions. Comprehensive mapping of pathways ensures effective coverage of programming to counter all possible paths to violence. By improving the systematic collection of information to a high standard, we can build more appropriate and targeted interventions to prevent violence against children.

## Supplementary Information

**Additional file 1.** List of literature repositories searched.

**Additional file 2.** Search strategy in Medline/PubMED.

**Additional file 3.** Risk of bias rating for included peer-reviewed articles and grey literature.

## Data Availability

The peer-reviewed and grey literature analyzed in this systematic review is publicly available online. Grey literature is available on the Humanitarian Response website, Save the Children Sweden – Resource Centre, the CP AoR website, IOM Online bookstore, and UNHCR’s online need assessments (https://www.humanitarianresponse.info/;https://resourcecentre.savethechildren.net; http://cpwg.net/resource-topics/assessment-3/;http://cpwg.net/starter-packs/;http://publications.iom.int/;http://needsassessment.unhcr.org/tools-and-templates/). All other remaining grey literature reports are available upon request from the lead agency or the CP AoR. Further requests for data may be directed to the corresponding author at it2208@caa.columbia.edu.

## Abbreviations

aOR: Adjusted Odds Ratio
CI: Confidence Interval
CP AoR: Child Protection Area of Responsibility
CPMS: Minimum Standards for Child Protection in Humanitarian Action
CRED: Centre for Research on the Epidemiology of Disasters
DV: Domestic violence
FGD: Focus group discussions
FGM: Female genital mutilation
IDMC: International Displacement Monitoring Centre
IFRC: International Federation of Red Cross and Red Crescent Societies
IOM: International Organization for Migration
IMO: Information Management Officer
PRISMA: Preferred Reporting Items for Systematic Reviews and Meta-Analyses
PTSD: Post-traumatic stress disorder
UNFPA: United Nations Population Fund
UNHCR: United Nations High Commissioner for Refugees
UNICEF: United Nations Children’s Fund
WASH: Water, Sanitation, and Hygiene
WHO: World Health Organization
