# Supplementary material for: The pathways between natural disasters and violence against children: a systematic review
Source: BMC Public Health. 2021 Jul 12;21:1249. doi: 10.1186/s12889-021-11252-3 (PMC8273959; doi:10.1186/s12889-021-11252-3)
Supplement: Supplementary file 1 — Additional file 1. List of literature repositories searched. [file 12889_2021_11252_MOESM1_ESM.docx]

**Additional file 1.** List of literature repositories searched

**Bibliographic databases**

| **Name** |
| --- |
| 1. MEDLINE/PubMED |
| 2. PsychINFO |
| 3. Global Health |
| 4. Social Policy and Practice |
| 5. Scopus |
| 6. Cumulative Index to Nursing & Allied Health Literature (CINAHL) Plus |
| 7. Africa-Wide Information |
| 8. Web of Science |
| 9. International Bibliography of Social Sciences (IBSS) |
| 10. Index Medicus for the Eastern Mediterranean Region (IMEMR) |
| 11. Western Pacific Region Index Medicus (WPRIM) |
| 12. Latin American and Caribbean Index Medicus (LILACS) |
| 13. MedCarib |
| 14. Indexing of Indian Medical Journals (IndMED) |
| 15. China Academic Literature Database (CNKI English) |

**Grey literature repositories**

| **Name** |
| --- |
| 1. Humanitarian Response  (Assessment reports; analysis reports; inter-agency humanitarian evaluation; and evaluation and lessons learned) |
| 2. Save the Children Sweden – Resource Centre  (Analysis; assessments; evaluations; reports; and studies, reviews, and research) |
| 3. Interagency Global Child Protection Area of Responsibility’s website  (Assessment reports and research) |
| 4. Interagency Global Child Protection Area of Responsibility’s website  (Child Protection Sub-Cluster Coordinator starter packs) |
| 5. IOM’s online bookstore  (International Migration Journal and studies and reports) |
| 6. UNHCR’s website  (Need assessments) |
