# Supplementary material for: The pathways between natural disasters and violence against children: a systematic review
Source: BMC Public Health. 2021 Jul 12;21:1249. doi: 10.1186/s12889-021-11252-3 (PMC8273959; doi:10.1186/s12889-021-11252-3)
Supplement: Supplementary file 2 — Additional file 2. Search strategy in Medline/PubMED. [file 12889_2021_11252_MOESM2_ESM.docx]

**Additional file 2.** Search strategy in Medline/PubMED

**1. Children**

|  | **Search terms** |
| --- | --- |
|  |  |
| 1 | child*.ab,ti. |
| 2 | minor*.ab,ti. |
| 3 | schoolchild*.ab,ti. |
| 4 | infan*.ab,ti. |
| 5 | adolescen*.ab,ti. |
| 6 | newborn*.ab,ti. |
| 7 | preschool*.ab,ti. |
| 8 | pre-school*.ab,ti. |
| 9 | kindergarten*.ab,ti. |
| 10 | underage.ab,ti. |
| 11 | youth.ab,ti. |
| 12 | youths.ab,ti. |
| 13 | baby.ab,ti. |
| 14 | babies.ab,ti. |
| 15 | prepubescen*.ab,ti. |
| 16 | pubescen*.ab,ti. |
| 17 | schoolage.ab,ti. |
| 18 | school-age.ab,ti. |
| 19 | boy*.ab,ti. |
| 20 | girl*.ab,ti. |
| 21 | offspring.ab,ti. |
| 22 | pediatric*.ab,ti. |
| 23 | paediatric*.ab,ti. |
| 24 | juvenile*.ab,ti. |
| 25 | toddler*.ab,ti. |
| 26 | “nursery school*”.ab,ti. |
| 27 | “elementary school*”.ab,ti. |
| 28 | “high school*”.ab,ti. |
| 29 | highschool*.ab,ti. |
| 30 | “primary school*”.ab,ti. |
| 31 | “secondary school*”.ab,ti. |
| 32 | daycare.ab,ti. |
| 33 | teen.ab,ti. |
| 34 | teens.ab,ti. |
| 35 | teenage*.ab,ti. |
| 36 | “child” [Mesh] |
| 37 | “adolescent” [Mesh] |
| 38 | “infant” [Mesh] |
| **39** | **OR/1-38** |

**2.** **Physical, emotional, or sexual violence**

| 40 | (physical adj2 violen*).ab,ti. |
| --- | --- |
| 41 | (sexual adj2 violen*).ab,ti. |
| 42 | (emotional adj2 violen*).ab,ti. |
| 43 | (psychological adj2 violen*).ab,ti. |
| 44 | (mental adj2 violen*).ab,ti. |
| 45 | (physical adj2 abuse).ab,ti. |
| 46 | (sexual adj2 abuse).ab,ti. |
| 47 | (emotional adj2 abuse).ab,ti. |
| 48 | (mental adj2 abuse).ab,ti. |
| 49 | (psychological adj2 abuse).ab,ti. |
| 50 | (verbal adj2 abuse).ab,ti. |
| 51 | “child abuse”.ab,ti. |
| 52 | (physical adj2 assault*).ab,ti. |
| 53 | (sexual adj2 assault*).ab,ti. |
| 54 | “physical attack*”.ab,ti. |
| 55 | (severe adj2 punishment).ab,ti. |
| 56 | (harsh adj2 punishment).ab,ti. |
| 57 | “corporal punishment”.ab,ti. |
| 58 | “corporeal punishment”.ab,ti. |
| 59 | (severe adj2 discipline).ab,ti. |
| 60 | (harsh adj2 discipline).ab,ti. |
| 61 | “harsh parenting”.ab,ti. |
| 62 | harass*ab,ti. |
| 63 | incest.ab,ti. |
| 64 | maltreat*.ab,ti. |
| 65 | “sexual violation*”.ab,ti. |
| 66 | “forced sex”.ab,ti. |
| 67 | “coerced sex”.ab,ti. |
| 68 | rape.ab,ti. |
| 69 | mistreat*.ab,ti. |
| 70 | molest*.ab,ti. |
| 71 | defile*.ab,ti. |
| 72 | (adverse adj childhood adj experience*).ab,ti. |
| 73 | “ACE”.ab,ti. |
| 74 | “violence against children”.ab,ti. |
| 75 | (abuse* adj2 spous*).ab,ti. |
| 76 | (abuse* adj2 partner*).ab,ti. |
| 77 | ((wife OR wives) adj2 abuse*).ab,ti. |
| 78 | ((wife OR wives) adj2 batter*).ab,ti. |
| 79 | (partner* adj2 violen*).ab,ti. |
| 80 | (spous* adj2 violen*).ab,ti. |
| 81 | “dating violen*”.ab,ti. |
| 82 | “bully*”.ab,ti. |
| 83 | “bullie*”.ab,ti. |
| 84 | ((antisocial OR agonis*) adj2 behavi*).ab,ti. |
| 85 | “intimidat*”.ab,ti. |
| 86 | “aggression*”.ab,ti. |
| 87 | (peer* adj2 violen*).ab,ti. |
| 88 | (peer* adj2 victim*).ab,ti. |
| 89 | “juvenile delinquency” [Mesh] |
| 90 | “bullying” [Mesh] |
| 91 | “physical abuse” [Mesh] |
| 92 | “domestic violence” [Mesh] |
| 93 | “intimate partner violence” [Mesh] |
| 94 | “spouse abuse” [Mesh] |
| 95 | “battered women” [Mesh] |
| 96 | “rape" [Mesh] |
| 97 | “child abuse” [Mesh] |
| 98 | **OR/40-97** |

**3. Natural disasters**

| 99 | (Natural adj2 disaster*).ti,ab. |
| --- | --- |
| 100 | (humanitarian adj2 (cris* OR emergenc* OR disaster*).ti,ab. |
| 101 | (complex adj2 emergenc*).ti,ab. |
| 102 | (displace$ adj2 (force$ OR population OR human OR internal$)).ti,ab. |
| 103 | avalanche*.ti,ab. |
| 104 | earthquake*.ti,ab. |
| 105 | temblor*.ti,ab. |
| 106 | volcano*.ti,ab. |
| 107 | "volcanic eruption*".ti,ab. |
| 108 | supervolcano*.ti,ab. |
| 109 | flood*.ti,ab. |
| 110 | landslide*.ti,ab. |
| 111 | mudslide*.ti,ab. |
| 112 | sinkhole*.ti,ab. |
| 113 | sink-hole*.ti,ab. |
| 114 | "limnic eruption*".ti,ab. |
| 115 | "tidal wave*".ti,ab. |
| 116 | tsunami*.ti,ab. |
| 117 | cyclone*.ti,ab. |
| 118 | "cyclonic storm*".ti,ab. |
| 119 | typhoon*.ti,ab. |
| 120 | hurricane*.ti,ab. |
| 121 | "tropical storm*".ti,ab. |
| 122 | tornado*.ti,ab. |
| 123 | storm*.ti,ab. |
| 124 | thunderstorm*.ti,ab. |
| 125 | rainstorm*.ti,ab. |
| 126 | hailstorm*.ti,ab. |
| 127 | "winter storm*".ti,ab. |
| 128 | snowstorm*.ti,ab. |
| 129 | blizzard*.ti,ab. |
| 130 | wildfire*.ti,ab. |
| 131 | bushfire*.ti,ab. |
| 132 | "heat wave*".ti,ab. |
| 133 | heatwave*.ti,ab. |
| 134 | drought*.ti,ab. |
| 135 | famine*.ti,ab. |
| 136 | "disasters" [MeSH] |
| 137 | **OR/99-136** |

**4. Combined terms**

| 138 | 39 AND 98 AND 137 |
| --- | --- |
| 139 | Limit 138 to (English language AND human) |
| 140 | 139 NOT ((case report OR editorial).ti,ab. OR editorial.ptyp. OR letter.ptyp. OR newspaper article.ptyp.) |
