# Supplementary material for: The pathways between natural disasters and violence against children: a systematic review
Source: BMC Public Health. 2021 Jul 12;21:1249. doi: 10.1186/s12889-021-11252-3 (PMC8273959; doi:10.1186/s12889-021-11252-3)
Supplement: Supplementary file 3 — Additional file 3. Risk of bias rating for included peer-reviewed articles and grey literature. [file 12889_2021_11252_MOESM3_ESM.docx]

**Additional file 3.** Risk of bias rating for included peer-reviewed articles and grey literature

**1. Qualitative peer-reviewed articles**

| Author | 1 | 2 | 3 | 4 | 5 | 6 | 7 | 8 | 9 | 10 | Quality score | |
| --- | --- | --- | --- | --- | --- | --- | --- | --- | --- | --- | --- | --- |
| Biswas et al. [15] | Y | Y | N | Not reported | N | N | N | N | Y | Y | 4 |  |
| Curtis  et al.  [50] | Y | N | N | Not reported | Not reported | N | Not reported | N | N | N | 1 |  |
| Davis and Bookey [53] | Y | Y | N | N | Not reported | N | N | N | N | N | 2 |  |
| Fisher [55] | Y | N | N | N | N | Not reported | Not reported | Not reported | Y | Y | 3 |  |
| Rashid and Michaud [49] | Y | Y | Y | Not reported | Y | Y | N | Y | Y | Y | 8 |  |
| Standing et al. [54] | Y | Y | N | N | N | N | N | N | Y | N | 3 |  |

**Legend:** Y = Yes and N = No. Quality categories: 1. Aims of research; 2. Appropriate method; 3. Appropriate research design; 4. Appropriate recruitment strategy; 5. Appropriate data collection; 6. Relationship between researcher and considered adequately; 7. Ethical issues considered; 8. Data analysis rigorous; 9. Statement of findings; 10. Valuable research

**2. Quantitative cohort and cross-sectional study design in peer-reviewed articles**

| Author | 1 | 2 | 3 | 4 | 5 | 6 | 7 | 8 | 9 | 10 | 11 | 12 | 13 | 14 | Quality score |
| --- | --- | --- | --- | --- | --- | --- | --- | --- | --- | --- | --- | --- | --- | --- | --- |
| Biswas et al.  [15] | Y | Y | Not reported | Y | Y | Y | Y | N | N | N | Y | N | N/A | Y | 8 |
| Catani et al.  [20] | Y | Y | Not  reported | Y | N | N | Not reported | Y | Y | N | Y | N | N/A | N | 6 |
| Scott  et al.  [52] | Y | Y | Y | Y | Y | N | N | Y | Y | N | Y | N | N/A | Y | 9 |

**Legend:** Y = Yes, N = No, and N/A = Not Applicable. Quality categories: 1. Clear research question; 2. Clear study population; 3. Participation rate of greater than or equal to 50%; 4. Recruited from the same population, timeframe, and consistent inclusion/exclusion; 5. Sample size justified, power description, or variance and effect estimation; 6. Exposure before outcome; 7. Timeframe under a year; 8 Varying levels of exposure measured; 9. Clear and consistent exposure; 10. Exposure assessed more than once over time; 11. Clear and consistent outcome; 12. Assessors blinded; 13. Loss to follow up less than or equal to 20%; 14. Key confounders adjusted

**3. Quantitative case-control study design in peer-reviewed articles**

| Author | 1 | 2 | 3 | 4 | 5 | 6 | 7 | 8 | 9 | 10 | 11 | 12 | Quality score |
| --- | --- | --- | --- | --- | --- | --- | --- | --- | --- | --- | --- | --- | --- |
| Terranova  et al.  [51] | Y | Y | N | N | N | Y | Y | Y | Y | Y | N/A | Y | 8 |

**Legend:** Y = Yes, N = No, and N/A = Not Applicable. 1. Clear research question; 2. Clear study population; 3. Sample size justified; 4. Same population and timeframe; 5. Consistent inclusion/exclusion; 6. Cases different from controls; 7. (if less than 100 cases or controls): random selection; 8. Concurrent controls; 9. Exposure before outcome; 10. Clear and consistent exposure; 11. Assessors blinded; 12. Key confounders adjusted or (if matching): proper statistics

**4. Qualitative grey literature**

| Author | 1 | 2 | 3 | 4 | 5 | 6 | 7 | 8 | 9 | 10 | Quality score |
| --- | --- | --- | --- | --- | --- | --- | --- | --- | --- | --- | --- |
| CARE Ethiopia Emergency Unit [71] | Y | Y | Y | Not reported | Not reported | N | N | N | Y | Y | 5 |
| Child Protection Sub-Cluster  [56] | Y | N | N | Y | Y | Not reported | Not reported | N | Y | Y | 5 |
| Civil Protection Zimbabwe  [70] | Y | Y | Y | Not reported | Not reported | N | N | N | Y | Y | 5 |
| Government of Bangladesh and Humanitarian Coordination Task Team of Bangladesh  [67] | Y | Y | Y | Y | Y | N | N | Y | Y | Y | 8 |
| International Federation of Red Cross and Red Crescent Societies [57] | Y | Y | Y | N | Y | N | N | Y | Y | Y | 7 |
| Jinks and Komenji [58] | Not reported | N | Not reported | Not reported | Not reported | Not reported | Not reported | N | Y | N | 1 |
| Ministry of International Affairs of the Government of the Kingdom of Tonga and Pacific Humanitarian Cluster [61] | Y | Y | Y | Not reported | Y | Not reported | Not reported | Not reported | Y | Y | 6 |
| Oxfam and  CARE Ethiopia [69] | Y | Y | Y | Y | Y | N | N | Y | Y | Y | 8 |
| People in Need Czech Republic [62] | Y | Y | Y | Not reported | Y | N | N | N | Y | Y | 6 |
| People in Need Czech Republic [63] | Y | Y | Y | Y | Y | N | N | N | N | Y | 6 |
| Plan International et al.  [64] | Y | Y | Y | Y | Y | N | N | Y | Y | Y | 8 |
| Polack [59] | Y | Y | Not reported | Not reported | Not reported | N | N | N | Y | Y | 4 |
| Save the Children [65] | Y | Y | N | N | N | N | N | N | Y | Y | 4 |
| Save the Children [72] | Y | Y | N | Not reported | Not reported | N | N | N | Y | Y | 4 |
| Save the Children [66] | Y | Y | Y | Not reported | Y | N | N | N | Y | Y | 6 |
| Save the Children [68] | Y | Y | Y | Not reported | Y | N | N | N | Y | Y | 6 |
| United Nations Population Fund and Ministère à la Condition féminine et aux Droits des femmes [60] | Y | Y | N | Not reported | Not reported | Not reported | N | N | Y | Y | 4 |

**Legend:** Y = Yes and N = No. 1. Aims of research; 2. Appropriate method; 3. Appropriate research design; 4. Appropriate recruitment strategy; 5. Appropriate data collection; 6. Relationship between researcher and considered adequately; 7. Ethical issues considered; 8. Data analysis rigorous; 9. Statement of findings; 10. Valuable research
